# Mobile Electronic Patient-Reported Outcomes and Interactive Support During Breast and Prostate Cancer Treatment: Health Economic Evaluation From Two Randomized Controlled Trials

**DOI:** 10.2196/53539

**Published:** 2025-03-11

**Authors:** Marie-Therése Crafoord, Joakim Ekstrand, Kay Sundberg, Marie I Nilsson, Maria Fjell, Ann Langius-Eklöf

**Affiliations:** 1 Division of Nursing Department of Neurobiology, Care Sciences and Society Karolinska Institutet Stockholm Sweden; 2 Faculty of Health Science Kristianstad University Kristianstad Sweden; 3 Function Area Social Work in Health Care Karolinska University Hospital Stockholm Sweden; 4 Academic Primary Care Centre Region Stockholm Stockholm Sweden

**Keywords:** cost-effectiveness, ePRO, mHealth, disease monitoring, cancer, RCT, randomized controlled trial, controlled trials, digital intervention, patient-reported outcomes, management, payers' perspective, health care costs, apps, prostate cancer, breast cancer

## Abstract

**Background:**

Digital interventions for supportive care during cancer treatment incorporating electronic patient-reported outcomes (ePROs) can enhance early detection of symptoms and facilitate timely symptom management. However, economic evaluations are needed.

**Objective:**

This study aims to conduct a cost-utility analysis of an app for ePRO and interactive support from the perspective of the payer (Region Stockholm Health Care Organization) and to explore its impact on patient health care utilization and costs.

**Methods:**

Two open-label randomized controlled trials (RCTs) were conducted, including patients undergoing neoadjuvant chemotherapy for breast cancer (B-RCT; N=149) and radiotherapy for prostate cancer (P-RCT; N=150), recruited from oncology clinics at 2 university hospitals in Stockholm, Sweden. EORTC QLQ-C30 scores were mapped to EQ-5D-3L to calculate quality-adjusted life years (QALYs). Intervention and implementation costs and health care costs, obtained from an administrative database, were used to calculate incremental cost-effectiveness ratios (ICERs) in 3 ways: including all health care costs (ICERa), excluding nonacute health care costs (ICERb), and excluding health care costs altogether (ICERc). Nonparametric bootstrapping was used to explore ICER uncertainty. Health care costs were analyzed by classifying them as disease-related or acute.

**Results:**

In both RCT intervention groups, fewer QALYs were lost compared with the control group (*P*<.001). In the B-RCT, the mean intervention cost was €92 (SD €2; €1=US $1.03). The mean cost for the intervention and all health care was €36,882 (SD €1032) in the intervention group and €35,427 (SD €959) in the control group (*P*<.001), with an ICERa of €202,368 (95% CI €152,008-€252,728). The mean cost for the intervention and acute health care was €3585 (SD €480) in the intervention group and €3235 (SD €494) in the control group (*P*<.001). ICERb was €49,903 (95% CI €37,049-€62,758) and ICERc was €13,213 (95% CI €11,145-€15,281); 22 out of 74 (30%) intervention group patients and 24 out of 75 (32%) of the control group patients required acute inpatient care for fever. In the P-RCT, the mean intervention cost was €43 (SD €0.2). The mean cost for the intervention and all health care was €3419 (SD €739) in the intervention group and €3537 (SD €689) in the control group (*P*<.001), with an ICERa of –€1,092,136 (95% CI –€3,274,774 to €1,090,502). The mean cost for the intervention and acute health care was €1219 (SD €593) in the intervention group and €802 (SD €281) in the control group (*P*<.001). ICERb was €745,987 (95% CI –€247,317 to €1,739,292) and ICERc was €13,118 (95% CI –68,468 to €94,704). As many as 10 out of the 75 (13%) intervention group patients had acute inpatient care, with the most common symptom being dyspnea, while 9 out of the 75 (12%) control group patients had acute inpatient care, with the most common symptom being urinary tract infection.

**Conclusions:**

ePRO and interactive support via an app generated a small improvement in QALYs at a low intervention cost and may be cost-effective, depending on the costs considered. Considerable variability in patient health care costs introduced uncertainty around the estimates, preventing a robust determination of cost-effectiveness. Larger studies examining cost-effectiveness from a societal perspective are needed. The study provides valuable insights into acute health care utilization during cancer treatment.

**Trial Registration:**

ClinicalTrials.gov NCT02479607; https://clinicaltrials.gov/ct2/show/NCT02479607, ClinicalTrials.gov NCT02477137; https://clinicaltrials.gov/ct2/show/NCT02477137

**International Registered Report Identifier (IRRID):**

RR2-10.1186/s12885-017-3450-y

## Introduction

Managing symptoms during cancer treatment is essential for patients’ quality of life, workability, and performance [1]. Symptoms such as pain, fatigue, and gastrointestinal problems commonly lead to emergency department visits [2]. Emergency department visit rates appear to be higher among patients with cancer than in the general population, although the magnitude or underlying reasons for this remain understudied [3].

Electronic patient-reported outcome (ePRO) interventions have gained recognition as convenient and safe tools for promoting the early detection of symptoms and adverse events [4,5]. Collecting ePROs has demonstrated high acceptance [6-8], long-term feasibility [9], and positive outcomes related to physical and psychological symptoms [10-15], as well as increased survival [16]. ePROs are also suggested to help mitigate unplanned acute care and unnecessary hospitalizations during cancer treatment; however, this assertion requires more robust empirical confirmation [17,18]. In our studies, the use of the interactive app Interaktor was associated with a decreased symptom burden during radiotherapy (RT) for prostate cancer [19], neoadjuvant chemotherapy (NACT) for breast cancer [11], and up to 6 months after surgery for pancreatic cancer [20].

Health economic evaluations are essential for supporting the implementation of cost-effective interventions [21,22] and guiding decision-makers [23]. A cost-utility analysis (CUA) is one type of health economic evaluation that compares the costs and health outcomes of medical treatments or care by estimating the cost required to improve a unit of health outcome [24]. Quality-adjusted life years (QALYs) is a generic measure of disease burden that accounts for both life quality and quantity. One QALY corresponds to 1 year of perfect health, while 0 represents death [25]. In Sweden, the National Board of Health and Welfare (NBHW) has defined a cost per QALY of €9685 (€1=US $1.03) as low, more than €48,423 as high, and more than €96,846 as very high [26].

Most health economic evaluations of ePRO interventions have focused on patients with advanced or metastatic cancer [27]. Lizée et al [28] demonstrated the cost-effectiveness of ePRO from a national health insurance perspective, despite increased costs, due to associated survival benefits. Velikova et al [29] evaluated the cost-effectiveness of ePRO for patients undergoing systemic treatment for colorectal, breast, or gynecological cancer, comparing the cost per additional QALY gained at 18 weeks after randomization from both health care and societal perspectives. The analysis considered costs for the intervention manual, software maintenance, and patient time off work but excluded intervention development costs. No significant cost differences were observed between the intervention and usual care groups. The study indicated a 55% likelihood of cost-effectiveness at the National Institute for Health and Care Excellence cost-per-QALY threshold.

This study was conducted alongside 2 randomized controlled trials (RCTs) of the ePRO intervention Interaktor during NACT for breast cancer (B-RCT) and RT for prostate cancer (P-RCT). The primary aim is to evaluate the cost utility of the app for ePRO and interactive support from the health care provider’s perspective (Region Stockholm Health Care Organization). Additionally, the study examines the impact on patients’ health care utilization and associated costs.

## Methods

### Ethical Considerations

The research was approved by the Swedish Ethical Review Authority (permit numbers 2013/1652-31/2 and 2017/2519-32). Written informed consent was obtained from all patients at the time of study inclusion. Data were deidentified to protect participants’ privacy. Patients received written and verbal information about their right to opt out without affecting their subsequent care. No compensation or payment was provided for participation.

### Study Design

Between 2016 and 2019, Interaktor was evaluated through 2 parallel prospective open-label RCTs, with symptom burden as the primary endpoint, measured using the EORTC QLQ-C30 version 3.0 [30]. Patients were consecutively recruited from oncology clinics at 2 university hospitals in Stockholm, Sweden. Eligible and interested patients met with a researcher who provided detailed information about the trial. Refer to the previously published study protocol and clinical effectiveness article [11,31] for details on the eligibility criteria, intervention design, and randomization process. No changes were made to the methods after the protocol was registered (NCT02479607 and NCT02477137) and the trials commenced.

### Samples

One RCT included a sample of patients with breast cancer treated with NACT (B-RCT), and the other included a sample of patients with prostate cancer treated with RT (P-RCT). In both RCTs, patients were randomly allocated to the intervention or control group. In the B-RCT, there were 74 patients in the intervention group and 75 patients in the control group. Of these, 69 (93.2%) in the intervention group and 71 (94.7%) in the control group completed the follow-up and were considered complete cases (Multimedia Appendices 1 and 2). In the P-RCT, a total of 150 patients were randomly allocated to the intervention (n=75) or control (n=75) group. Of these, 58 (77%) in the intervention group and 56 (75%) in the control group completed the follow-up questionnaires and were considered complete cases [32] (Multimedia Appendices 2-4). The sample size for both RCTs was estimated based on an effect study conducted with patients receiving RT for prostate cancer [19], with symptom distress as the primary outcome. The effect size difference (Cohen *d*=0.54) indicated that, for 90% power at *P*<.05, 71 patients were required in each group.

### Intervention and Standard Care

The Interaktor smartphone and tablet app is an ePRO intervention designed for daily symptom reporting and interactive support during cancer treatment. It includes a symptom questionnaire, graphs of symptom reporting history, self-care advice related to disease and treatment-associated symptoms, and links to websites with additional information. Oncology ward nurses are alerted via SMS text messages when severe symptom levels are reported. Nurses can access patients’ reports through a web interface, which facilitates patient-clinician communication. Depending on the alert, nurses contact the patient within 1 hour or 1 day. The Interaktor versions used in this study did not include any institutional affiliation display or logo.

Patients in the intervention group reported daily via the Interaktor app on weekdays, starting from their first day of treatment and continuing until 2 weeks after treatment in the B-RCT (mean treatment duration: 15 weeks in both groups) and, until 3 weeks after treatment in the P-RCT (mean treatment duration: 5 weeks in both groups). In the intervention groups, registered nurses at the patients’ oncology units responded to the symptom report alerts. Additionally, a researcher was available to assist with any technical questions or issues. Outside office hours, patients were advised to contact health care personnel according to the standard procedure of their oncology clinic. The intervention and app content remained unchanged during the evaluation process. Patients received daily reminders if a report had not been submitted. A comprehensive description, including screenshots, has been published previously [7].

All patients, in both the intervention and control groups, received standard care, which included an assigned contact nurse and a visit with the physician before treatment.

### Data Collection

Before randomization, patients self-reported sociodemographic characteristics, including education level, marital and occupational status, and baseline outcomes via questionnaires. In the B-RCT, follow-up (via postal questionnaires) occurred 2 weeks after the end of NACT or the day before surgery, whichever came first. In the P-RCT, follow-up was 3 weeks after the conclusion of RT. Medical history and clinical treatment data were obtained from the patients’ medical records, including comorbid conditions, tumor histopathology, cancer stage, and prostate-specific antigen score before treatment initiation, as well as the type and number of cancer treatments planned and completed, and reasons for discontinuing or altering treatment. Data on mortality and cause of death were obtained from the Stockholm and Gotland Regional Cancer Centre and the Swedish NBHW (Multimedia Appendix 5).

### Health Care Utilisation and Costs

Administrative data on each patient’s health care utilization and costs, from the first day of treatment and for 6 months thereafter, were obtained from the Stockholm Region Council administrative database (VAL). The database includes variables on primary care and emergency department visits (VAL-OVR) and hospitalizations (VAL-SLV) for Stockholm Region Council patients [33]. Health care costs were estimated using a variable (SIMKOST [simulerad kostnad/simulated cost]) that calculates the cost of visits based on the profit and loss account for the respective care branch. SIMKOST reflects approximately 90% of the costs for individuals’ visits to outpatient care and 99% of the costs for inpatient care (Multimedia Appendix 6). Intervention costs were based on a fiscal estimate provided by the company that developed the app, expressed as a 1-time implementation/startup cost of €5212, with weekly licensing costs per capita of €39 for nurses and €2.25 for patients (Multimedia Appendix 6).

### Data Analysis

#### Statistical Analysis

Data were handled using Microsoft Excel 2016 with the add-in XL-STAT, IBM SPSS Statistics version 27, and STATA 16 (StataCorp LP). Clinical trials with an RCT design should be analyzed using the intention-to-treat (ITT) principle [34], so missing values were imputed as the mean per group and time [32]. Health care utilization and cost values were imputed for 2 patients in each intervention group (B-RCT and P-RCT). In the P-RCT, EORTC dimension scores were imputed at baseline for 2 patients per group, and at follow-up for 15 patients in the intervention group and 20 patients in the control group. In the B-RCT, follow-up values were imputed for 5 patients in the intervention group and 4 patients in the control group. Distribution normality was assessed using skewness and kurtosis. All costs were adjusted for inflation from 2019 to 2022 [35] (×1.0764) and converted from Swedish kronor (SEK) to Euros (€) using the average exchange rate for April 2022 of 10.3257 SEK=€1 [36]. Nonparametric bootstrapping (1000 replications) was used to test nonnormally distributed variables, calculate the incremental cost-effectiveness ratios (ICERs), and explore sample uncertainty regarding the mean ICERs [37].

#### Health Outcome

EORTC QLQ-C30 [30] dimension scores were mapped onto EQ-5D-3L [38] health state utilities using a response mapping algorithm [38,39] (Multimedia Appendix 4). The original algorithm includes British utility weights [40], which were replaced with Swedish weights according to Burström et al [41] for this study. The mean predicted EQ-5D value (EQ-5DP) before treatment minus after treatment was used to measure effectiveness, with a smaller reduction in mean EQ-5DP indicating better outcomes.

#### Intervention Costs

The overall startup cost was divided by the total number of patients diagnosed and treated with the respective treatment regimens in the Stockholm Regional Council and Gotland Region for the years 2016-2018 (518 patients with breast cancer treated with NACT and 683 patients with prostate cancer treated with RT). Given that system updates may incur additional costs beyond the license fees, a time frame of 3 years was considered reasonable. The estimate assumed 5 nurses per 100 patients, with no additional costs for nurses to handle symptom alerts. Based on each patient’s number of weeks in treatment (wt), the intervention costs were calculated per equations (1) and (2) for B-RCT and P-RCT, respectively:

(5212/518) + ([39 × 5/100] × [wt]) + (2.25 × wt) **(1)**

(5212/683) + ([39 × 5/100] × [wt]) + (2.25 × wt) **(2)**

#### Cost-Utility Analysis

Stochastic CUAs [42] were conducted for each RCT by calculating ICERs in 3 different ways. For ICERa, each patient’s intervention cost, along with all health care costs from randomization through 6 months, was included. For ICERb, each patient’s intervention costs, plus acute health care costs from randomization and the subsequent 6 months, were considered. Given the considerable variation in patient health care costs, which introduced substantial uncertainty in the cost-effectiveness estimates, a third ICER (ICERc) was calculated by dividing the intervention group’s intervention costs minus the control group’s intervention costs by the difference in QALYs lost between the 2 groups. The rationale behind this approach is that patients’ health care utilization during cancer treatment is influenced by multiple factors, and a much larger study would be necessary to demonstrate a significant reduction in health care costs. Therefore, it was deemed appropriate to assess cost-effectiveness under the assumption that health care costs are not substantially affected. To capture the gradual change in the quality of life during treatment, QALYs lost were calculated linearly as follows: ([EQ-5DP after treatment minus EQ-5DP before treatment]/2) × (individual treatment duration in weeks/52). For visualization, bootstrap values of the incremental intervention costs and incremental health outcomes (QALYs) were plotted on cost-effectiveness planes.

In the P-RCT, the RT treatment was standardized with minimal variation between patients, so RT costs were excluded from both CUAs. By contrast, the B-RCT did not allow for standardized subtraction of treatment costs, so all health care costs were included. The analysis was conducted from the payer perspective (Stockholm Region Council) and focused on the patient’s treatment duration (less than 1 year), meaning that no discounting of costs or results was applied. The cost per QALY, as defined by the Swedish NBHW, was used to evaluate cost-effectiveness.

ICERa=[(intervention costs + IG total health care costs) – (CG total health care costs)]/(IG change in QALY – CG change in QALY)

ICERb=[(intervention costs + IG acute health care costs) – (CG acute health care costs)]/(IG change in QALY – CG change in QALY)

ICER=[(IG intervention costs) – (IG intervention costs)]/(IG change in QALY – CG change in QALY)

#### Exploration of Health Care Utilization and Costs

Within each RCT, variables for total and acute health care visits and costs were generated by summing each participant’s visits and costs, conditional on the VAL variable AKUT (acute) being marked as yes or no. Additionally, variables for health care utilization related to the respective cancer treatments were created through a qualitative analysis of the International Statistical Classification of Diseases and Related Health Problems (ICD) codes, using a conventional and summative approach [43]. All ICD codes for acute outpatient and inpatient visits within each RCT were compiled in Excel sheets, and the occurrence of all unique codes was counted. These ICD codes were either grouped or coded based on similarities into predefined and emerging categories. Examples of these categories include fever/neutropenia (D709C, R502, R508, and R509), gastroenteritis/colitis (K521 and A047), anemia (D649), urinary tract infection (N390), and urinary problems (R339, N390, R301, N390X, N304, N109, T830, R391, R319, and N300; Multimedia Appendix 7).

Each patient’s visits and costs, according to the categories, were calculated to create variables used as dependent outcomes in multivariate regression analysis. Depending on the level of overdispersion, Poisson, negative binomial, or binary logistic models with a log-link function were fitted [44]. The variable “Group” was coded as control=0 and intervention=1. Prior studies have suggested an association between diminished performance status [45,46], the presence of multiple chronic diseases in older individuals [47], and increased costs. Therefore, the continuous variables—age at inclusion, Charlson Comorbidity Score, and Baseline EQ-5DP score—were included as covariates. The reference category was arranged in ascending order. For the B-RCT, each patient’s number of NACT cycles was included as an independent variable. By contrast, for the prostate cancer trial, treatment was standardized, and all patients underwent a similar number of treatments.

## Results

### B-RCT

#### Health Outcome

The mean EQ-5DP before treatment was 0.86 in the intervention group and 0.87 in the control group. After treatment, the mean EQ-5DP was 0.84 in the intervention group and 0.80 in the control group (*P*=.036, effect size=0.099). A statistically significant difference was observed in the mean changes in EQ-5DP from before to after treatment between the intervention and control groups (*P*=.012, effect size=0.042). The greatest difference in change was observed in the Anxiety/Depression dimensions (Multimedia Appendix 8). The CONSORT (Consolidated Standards of Reporting Trials) checklist is presented in Multimedia Appendix 9 (also see Multimedia Appendices 1 and 3).

#### Cost-Utility Analysis

The intervention group patients had a mean QALY loss of –0.004 (SD 0.002) from before treatment to after treatment, while the corresponding figure for patients in the control group was –0.012 (SD 0.002; *P*<.001). The mean cost for the Interaktor app per patient was €92 (SD €2). The mean total cost for the intervention and all health care was €36,882 (SD €1032) for patients in the intervention group and €35,427 (SD €959) for control group patients (*P*<.001). The ICERa was €202,368 (€SD 811,136; 95% CI €152,008-€252,728). The mean cost for the intervention and acute health care was €3585 (SD €480) in the intervention group and €3235 (SD €494) in the control group (*P*<.001). ICERb was €49,903 (SD €207,042; 95% CI €37,049-€62,758; Table 1). Lastly, when health care costs were excluded from the analysis, the ICERc was €13,213 (SD €33,327; 95% CI €11,145-€15,281; Table 1).

**Table 1:** Breast cancer trial cost-utility analysis.

			Intervention group (n=74)		Control group (n=75)						
			Mean (SD)		Mean (SD)		*P* value		*t* test (*df*)^a^		95% CI
**Health utility**											
	QALYs^b,c^	–0.004 (0.002)		–0.012 (0.002)		<.001		80 (1998)		0.0074-0.0078	
	Incremental QALYs^b^	0.0076 (0.003)		N/A^d^		N/A		N/A		N/A	
**Costs (€^e^)**											
	Intervention costs	92 (2)		N/A		N/A		N/A		N/A	
**All health care costs (€)**											
	Outpatient	27,571 (6392)		26,348 (5800)		N/A		N/A		N/A	
	Inpatient	9207 (5254)		9093 (5460)		N/A		N/A		N/A	
	Total	36,882 (1032)		35,427 (959)		<.001		33 (1987)		33,530-37,351	
	Incremental^b^	1454 (1386)		N/A		N/A		N/A		N/A	
**Acute health care costs (€)**											
	Outpatient	554 (597)		562 (606)		N/A		N/A		N/A	
	Inpatient	2932 (4023)		2665 (3992)		N/A		N/A		N/A	
	Total	3585 (480)		3235 (494)		<.001		16 (1998)		2242-4214	
	Incremental^b^	353 (676)		N/A		N/A		N/A		N/A	
ICERa^b,f^			202,368 (811,136)		N/A		N/A		N/A		152,008-252,728
ICERb^b^			49,903 (207,042)		N/A		N/A		N/A		37,049-62,758
ICERc^b^			13,213 (33,327)		N/A		N/A		N/A		11,145-15,281

^a^Independent unpaired samples Student *t* test (2-tailed).

^b^Based on bootstrap.

^c^QALY: quality-adjusted life year.

^d^N/A: not applicable.

^e^€1=US $1.03.

^f^ICER: incremental cost-effectiveness ratio.

**Figure 1:**
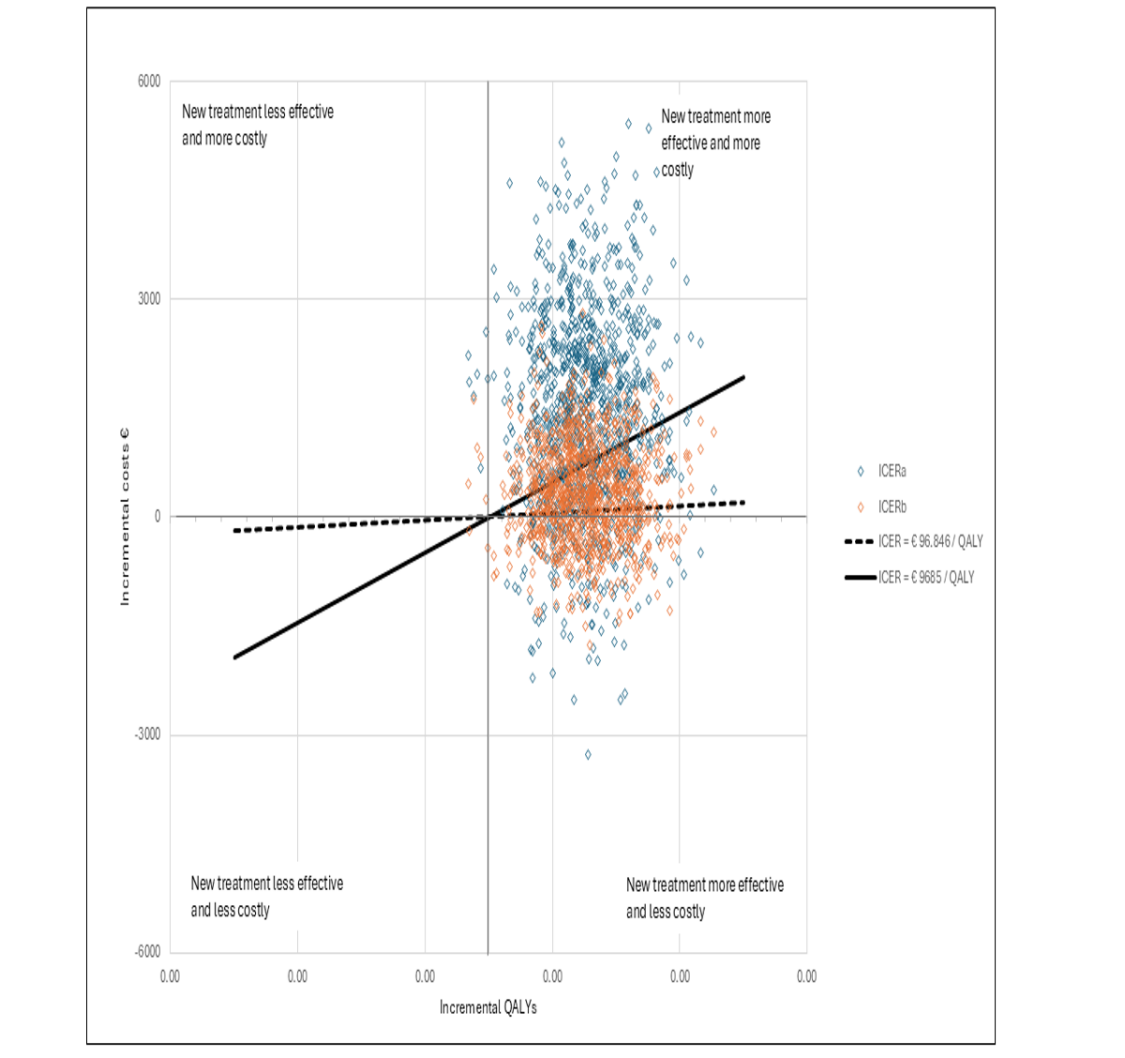
Breast cancer cost-effectiveness plane ICERa and ICERb. ICER: incremental cost-effectiveness ratio; QALY: quality-adjusted life year.

**Figure 2:**
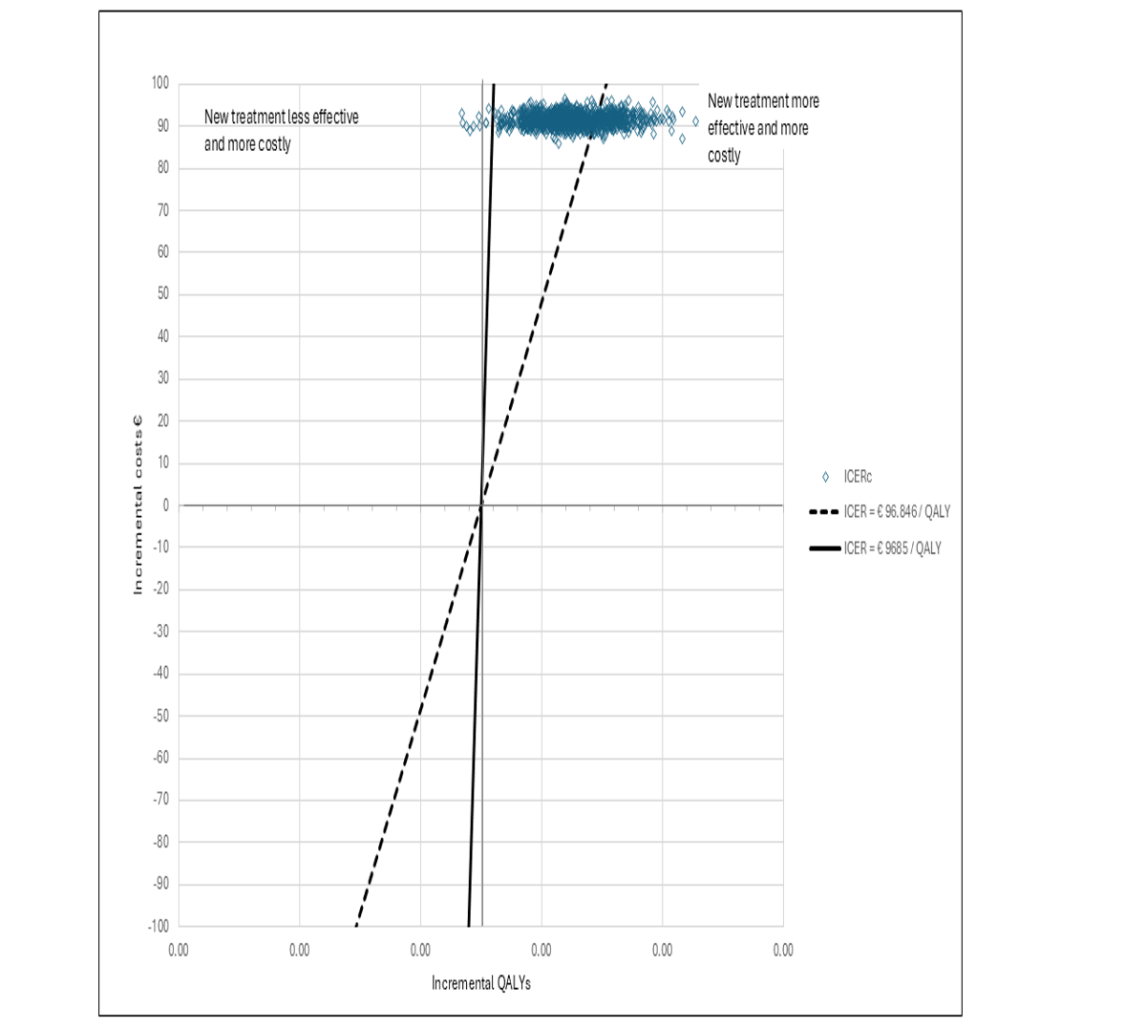
Breast cancer cost-effectiveness plane ICERc. ICER: incremental cost-effectiveness ratio; QALY: quality-adjusted life year.

Figure 1 presents a cost-effectiveness plane depicting the bootstrapped values of the intervention group’s joint incremental costs and incremental QALYs compared with the control group, as per ICERa and ICERb. Figure 2 illustrates the cost-effectiveness plane with the corresponding values based on ICERc.

#### Exploration of Health Care Utilization and Health Care Costs

The mean outpatient cost for patients in the intervention group was €27,571 (SD €6392), while in the control group, it was €26,348 (SD €5800). In both groups, approximately 2% of the outpatient costs were attributable to acute care: €554 out of €29,321 (1.9%) in the intervention group and €562 out of €26,348 (2.13%) in the control group.

In the intervention group, 13 out of 74 (18%) patients had an acute outpatient visit for fever, with a total of 34 visits. In the control group, the corresponding proportion was 9 out of 75 (12%), with a total of 21 visits. Additionally, 7 out of 74 (9%) patients in the intervention group had an unplanned admission from outpatient to inpatient care, accounting for 37 unplanned admissions. In the control group, 6 out of 75 (8%) had an unplanned admission from outpatient to inpatient care, totaling 29 unplanned admissions.

The mean inpatient cost per patient was €9207 (SD €5254) in the intervention group and €9093 (SD €5460) in the control group. Approximately one-third of all inpatient care cost was acute in both groups (€2932/€9207, 31.85% in the intervention group and €2665/€9093, 29.31% in the control group). The most common diagnoses during acute inpatient care episodes in both groups were fever, gastroenteritis/colitis, anemia, and urinary tract infection. The variable group (intervention/control) was not associated with the number of visits for fever, gastroenteritis/colitis, anemia, or urinary tract infection, nor were age, health-related quality of life (HRQOL) before treatment, comorbidities, or the number of NACT (Table 2).

**Table 2:** Breast cancer trial multivariate regression analysis of predictors for acute healthcare visits for chemotherapy-related symptoms

				Intervention group (n=74)	Control group (n=75)	B	SE	Standardized coefficient [Exp(B)]	95% CI	*P* value	*χ*^2^/*df*^a^
**Visits**											
	**Acute outpatient**										
		Fever^b^ M (SD)		0.46 (0.86)	0.28 (0.63)	N/A^c^	N/A	N/A	N/A	N/A	N/A
		Dependent variable: total, n		34 (n=13)	21 (n=9)	N/A	N/A	N/A	N/A	N/A	N/A
		Independent variables:		N/A	N/A	N/A	N/A	N/A	N/A	.49^d^	1.141
			Group^e^	N/A	N/A	–0.46	0.33	0.63	0.331-1.202	.16^f^	N/A
			Age	N/A	N/A	–0.01	0.02	0.992	0.949-1.037	.72^f^	N/A
			HRQOL	N/A	N/A	–0.70	1.93	0.498	0.011-22.106	.72^f^	N/A
			Comorbidity	N/A	N/A	0.15	0.20	1.156	0.773-1.727	.48^f^	N/A
			NACT^g^	N/A	N/A	–0.08	0.07	0.92	0.800-1.058	.24^f^	N/A
		Unplanned admissions M (SD)		0.50 (71)	0.39 (0.72)	N/A	N/A	N/A	N/A	N/A	N/A
		Dependent variable: total, n		37 (n=7)	29 (n=6)	N/A	N/A	N/A	N/A	N/A	N/A
		Independent variables		N/A	N/A	N/A	N/A	N/A	N/A	.52^d^	.877
			Group^e^	N/A	N/A	–0.220	0.302	0.803	0.444-1.451	.47^f^	N/A
			Age	N/A	N/A	0.010	0.021	1.010	0.969-1.054	.62^f^	N/A
			HRQOL	N/A	N/A	–3.048	1.760	0.047	0.002-1.494	.08^f^	N/A
			Comorbidity	N/A	N/A	0.029	0.193	1.030	0.705-1.504	.88^f^	N/A
			NACT^g^	N/A	N/A	–0.036	0.061	0.965	0.857-1.086	.55^f^	N/A
	**Acute inpatient**										
		All M (SD)		0.73 (0.98)	0.60 (0.82)	N/A	N/A	N/A	N/A	N/A	N/A
		Dependent variable: total, n		54 (n=35)	45 (n=32)	N/A	N/A	N/A	N/A	N/A	N/A
		Independent variables:		N/A	N/A	N/A	N/A	N/A	N/A	.72^d^	.722
			Group^e^	N/A	N/A	–0.182	0.2636	0.834	0.497-1.398	.49^f^	N/A
			Age	N/A	N/A	0.006	0.0187	1.006	0.969-1.043	.76^f^	N/A
			HRQOL	N/A	N/A	–1.291	1.634	0.275	0.011-6.768	.43^f^	N/A
			Comorbidity	N/A	N/A	0.055	0.1702	1.057	0.757-1.476	.74^f^	N/A
			NACT^g^	N/A	N/A	–0.068	0.0568	0.934	0.836-1.044	.23^f^	N/A
		Fever^b^ M (SD)		0.39 (0.70)	0.32 (0.55)	N/A	N/A	N/A	N/A	N/A	N/A
		Dependent variable: Total, n		29 (n=22)	24 (n=21)	N/A	N/A	N/A	N/A	N/A	N/A
		Independent variables:		N/A	N/A	N/A	N/A	N/A	N/A	.94^d^	.838
			Group^e^	N/A	N/A	–0.20	0.32	0.815	0.433-1.535	.53^f^	N/A
			Age	N/A	N/A	0.01	0.02	1.01	0.965-1.057	.66^f^	N/A
			HRQOL	N/A	N/A	–1.04	1.94	0.353	0.008-15.75	.59^f^	N/A
			Comorbidity	N/A	N/A	–0.13	0.23	0.876	0.557-1.378	.57^f^	N/A
			NACT^g^	N/A	N/A	–0.01	0.07	0.988	0.868-1.124	.86^f^	N/A
		Gastroenteritis^b^ M (SD)		0.08 (0.36)	0.16 (0.40)	N/A	N/A	N/A	N/A	N/A	N/A
		Dependent variable: total, n		6 (n=4)	12 (n=11)	N/A	N/A	N/A	N/A	N/A	N/A
		Independent variables:		N/A	N/A	N/A	N/A	N/A	N/A	.77^d^	1.186
			Group^e^	N/A	N/A	0.66	0.53	1.931	0.686-5.440	.21^f^	N/A
			Age	N/A	N/A	0.02	0.04	1.019	0.944-1.100	.63^f^	N/A
			HRQOL	N/A	N/A	1.83	3.35	6.245	0.009-4422.840	.58^f^	N/A
			Comorbidity	N/A	N/A	–0.31	0.41	0.737	0.328-1.657	.46^f^	N/A
			NACT^g^	N/A	N/A	0.004	0.100	1.004	0.826-1.220	.97^f^	N/A
		Anemia^b^ M (SD)		0.05 (0.23)	0.05 (0.23)	N/A	N/A	N/A	N/A	N/A	N/A
		Dependent variable: total, n		4 (n=4)	4 (n=4)	N/A	N/A	N/A	N/A	N/A	N/A
		Independent variables:		N/A	N/A	N/A	N/A	N/A	N/A	.73^d^	.961
			Group^e^	N/A	N/A	-0.10	0.74	0.901	0.210-3.863	.89^f^	N/A
			Age	N/A	N/A	0.10	0.07	1.101	0.963-1.258	.16^f^	N/A
			HRQOL	N/A	N/A	1.85	4.74	6.346	0.001-68734.141	.70^f^	N/A
			Comorbidity	N/A	N/A	-0.94	0.73	0.389	0.093-1.633	.20^f^	N/A
			NACT^g^	N/A	N/A	0.03	0.14	0.029	0.780-1.357	.89^f^	N/A
		Urinary tract infection^b^ M (SD)		0.04 (0.20)	0.05 (0.03)	N/A	N/A	N/A	N/A	N/A	N/A
		Dependent variable: total, n		3 (n=3)	4 (n=3)	N/A	N/A	N/A	N/A	N/A	N/A
		Independent variables:		N/A	N/A	N/A	N/A	N/A	N/A	.31^d^	1.268
			Group^e^	N/A	N/A	–0.09	0.84	0.911	0.177-4.702	.91^f^	N/A
			Age	N/A	N/A	0.03	0.05	1.030	0.926-1.146	.58^f^	N/A
			HRQOL	N/A	N/A	4.01	6.39	55.11	0.000- 15250488	.53^f^	N/A
			Comorbidity	N/A	N/A	0.29	0.43	1.34	0.571-3.144	.50^f^	N/A
			NACT^g^	N/A	N/A	0.07	0.14	1.08	0.818-1.426	.59^f^	N/A

^a^Pearson χ2 value divided by degrees of freedom (goodness of fit of the model).

^b^Nr of acute visits when a patient received the diagnose.

^c^N/A: not applicable.

^d^*P* value Omnibus test General Linear Model Negative Binomial Regression.

^e^Intervention/Control; Reference category=Intervention

^f^*P* value for the independent variable in the General Linear Model Negative Binomial Regression.

^g^Neoadjuvant chemotherapy treatments

Negative binomial multivariate regression analysis revealed that the independent variable group (intervention/control) did not significantly affect the predicted log odds of patients’ health care costs (*P*=.949). For acute outpatient health care costs, the analysis showed that age, health-related quality of life at baseline, and comorbidities significantly predicted costs. Older age (*P*=.002) and better health-related quality of life (*P*<.001) were associated with lower acute outpatient health care costs. By contrast, a higher number of comorbidities was associated with increased acute health care costs (*P*=.02; Table 3).

**Table 3:** Breast cancer trial multivariate regression analysis of predictors for health care costs.

					Intervention group (n=74)		Control group (n=75)		Unstandardized coefficient		SE		Standardized coefficient [Exp(B)]		95% CI		*P* value		*χ*^2^/*df*^a^
**Care costs (€^b^)**																			
	**Outpatient**																		
		All costs Mean (SD)			27,571 (6392) n=74		26,348 (5800) n=75		N/A^c^		N/A		N/A		N/A		N/A		N/A
		Dependent variable: Total costs			2,040,225		1,976,097		N/A		N/A		N/A		N/A		.95^d^		0.042
		Independent variables:																	
			Group^e^	N/A		N/A		–0.038		0.166		0.963		0.695-1.334		.82^f^		N/A	
			Age	N/A		N/A		–0.001		0.012		0.999		0.976-1.023		.94^f^		N/A	
			HRQOL	N/A		N/A		–0.78		1.019		0.458		0.062-3.375		.44^f^		N/A	
			Comorbidity	N/A		N/A		–0.012		0.115		0.988		0.789-1.239		.92^f^		N/A	
			NACT^g^	N/A		N/A		0.014		0.033		1.014		0.951-1.081		.67^f^		N/A	
		Acute costs Mean (SD)			554 (597) n=51		562 (606) n=52		N/A		N/A		N/A		N/A		N/A		N/A
		Dependent variable: Acute costs			40,998		42,183		N/A		N/A		N/A		N/A		<.001^d^		1.170^a^
		Independent variables:																	
			Group^e^	N/A		N/A		0.043		0.166		1.044		0.754-1.446		.80^f^		N/A	
			Age	N/A		N/A		–0.036		0.012		0.964		0.942-0.987		.002^f^		N/A	
			HRQOL	N/A		N/A		–4.091		1.108		0.017		0.002-0.147		<.001^f^		N/A	
			Comorbidity	N/A		N/A		0.247		0.110		1.280		1.033-1.588		.02^f^		N/A	
			NACT^g^	N/A		N/A		–0.062		0.032		0.940		0.882-1.001		.05^f^		N/A	
	**Inpatient**																		
		All costs Mean (SD)			9206 (5254) n=69		9093 (5460) n=73		N/A		N/A		N/A		N/A		N/A		N/A
		Dependent variable: Total costs			681,221		681,949		N/A		N/A		N/A		N/A		.84^d^		0.341^a^
		Independent variables:																	
			Group^e^	N/A		N/A		0.008		0.165		1.008		0.73-1.393		.96^f^		N/A	
			Age	N/A		N/A		–0.004		0.012		0.996		0.973-1.018		.70^f^		N/A	
			HRQOL	N/A		N/A		–1.041		1.044		0.353		0.046-2.733		.32^f^		N/A	
			Comorbidity	N/A		N/A		–0.011		0.111		0.989		0.796-1.229		.92^f^		N/A	
			NACT^g^	N/A		N/A		–0.01		0.033		0.990		0.928-1.056		.77^f^		N/A	

^a^€1=US $1.03.

^b^N/A: not applicable.

^c^*P* value Omnibus test General Linear Model Multivariate Regression.

^d^Pearson χ2 value divided by degrees of freedom (goodness of fit of the model).

^e^Intervention/Control; Reference category=Intervention

^f^*P* value for the independent variables in the General Linear Model Multivariate Regression Model.

^g^Neoadjuvant chemotherapy treatments

### P-RCT

#### Health Outcome

The mean EQ-5DP before treatment was 0.88 in the intervention group and 0.89 in the control group. After treatment, the mean EQ-5DP was 0.87 in the intervention group and 0.88 in the control group (*P*=.51). The mean difference in EQ-5DP from before to after treatment was not statistically significant between the intervention and control groups (*P*=.94). The most prominent differences in change were observed in the dimensions of Pain/Discomfort and Anxiety/Depression (Multimedia Appendix 5).

#### Cost-Utility Analysis

The intervention group patients scored a mean QALY loss of –0.0008 (SD 0.0006), while the corresponding figure for the control group was –0.0009 (SD 0.0006; *P*<.001). The mean total cost of the Interaktor intervention per patient was €43 (SD €0.2). The mean total cost, including the intervention and all health care, was €3419 (SD €739) for the intervention group and €3537 (SD €689) for the control group. The ICERa was –€1,092,136 (SD €35,155,229; 95% CI –€3,274,774 to –€1,090,502). The mean total costs for the intervention and acute health care were €1219 (SD €593) in the intervention group and €802 (SD €281) in the control group. The ICERb was €745,987 (SD €16,006,924; 95% CI –€247,317 to €1,739,292). Lastly, when health care costs were excluded from the analysis, the ICERc was €13,118 (SD €1,314,743; 95% CI –€68,468 to €94,704; Table 4).

**Table 4:** Prostate cancer trial cost-utility analysis.

			Intervention group (n=75)		Control group (n=75)						
			Mean (SD)		Mean (SD)		*P* value^a^		*t* test (*df*)		95% CI
**Health utility**											
	QALYs^b,c^	–0.0008 (0.0006)		–0.0009 (0.0006)		<.0001		6.419 (1998)		0.0001 to 0.0002	
	Incremental QALYs^b^	0.0002 (0.0008)		N/A^d^		N/A		N/A		N/A	
**Costs (€^e^)**											
	Intervention costs	43 (0.2)		N/A		N/A		N/A		N/A	
**All health care costs^f^ (€)**											
	Outpatient	2077 (1386)		2488 (2403)		N/A		N/A		N/A	
	Inpatient	1321 (5460)		1049 (4240)		N/A		N/A		N/A	
	Total	3419 (739)		3537 (689)		.0002		–4 (1988)		–183 to –57	
	Incremental^b^	–120 (1034)		N/A		N/A		N/A		N/A	
**Acute health care costs (€)**											
	Outpatient	121 (247)		126 (258)		N/A		N/A		N/A	
	Inpatient	1054 (5132)		684 (2335)		N/A		N/A		N/A	
	Total	1219 (593)		802 (281)		<.0001		20 (1426)		376 to 458	
	Incremental^b^	417 (659)		N/A		N/A		N/A		N/A	
ICERa^b^			–1,092,136 (35,155,229)		N/A		N/A		N/A		–3,274,774 to 1,090,502
ICERb^b^			745,987 (16,006,924)		N/A		N/A		N/A		–247,317 to 1,739,292
ICERc^b^			13,118 (1,314,743)		N/A		N/A		N/A		–68,468 to 94,704

^a^Independent unpaired samples Student t test (2-tailed).

^b^Based on bootstrap.

^c^QALY: quality-adjusted life year.

^d^N/A: not applicable.

^e^€1=US $1.03.

^f^Excluding radiotherapy costs.

gICER: incremental cost-effectiveness ratio.

**Figure 3:**
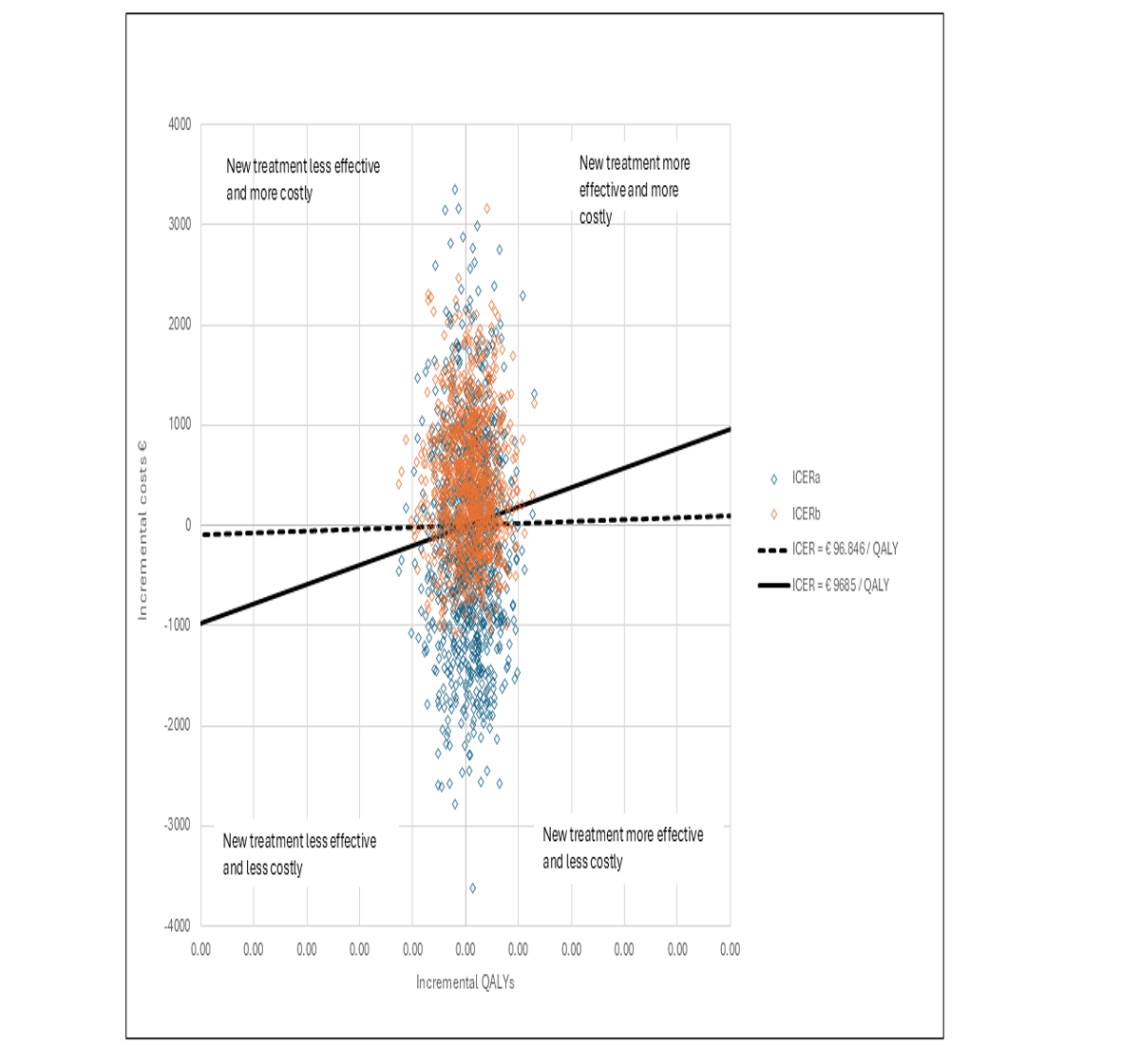
Prostate cancer cost-effectiveness plane ICERa and ICERb. ICER: incremental cost-effectiveness ratio; QALY: quality-adjusted life year.

**Figure 4:**
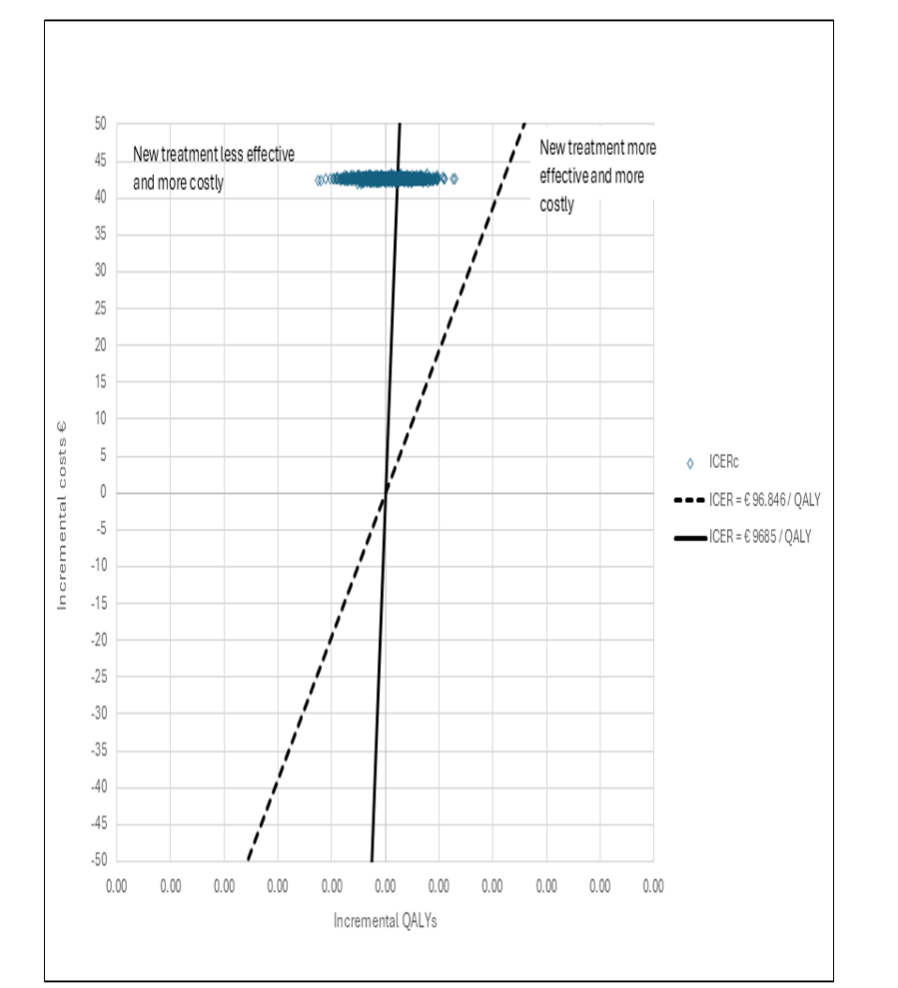
Prostate cancer cost-effectiveness plane ICERc. ICER: incremental cost-effectiveness ratio; QALY: quality-adjusted life year.

Figure 3 presents a cost-effectiveness plane showing the bootstrapped values of the intervention group’s joint incremental costs and incremental QALYs compared with the control group for ICERa and ICERb. Figure 4 displays the cost-effectiveness plane with the corresponding values for ICERc.

#### Exploration of Health Care Utilization and Health Care Costs

The mean outpatient cost for patients in the intervention group was €2077 (SD €1386), while in the control group, it was €2488 (SD €2403); €121 out of €2077 (5.83%) outpatient cost in the intervention group was for acute care, compared with €126 out of €2488 (5.06%) in the control group. In both groups, 25 patients (33%) had an acute outpatient care visit.

Acute outpatient care for urological problems was required by 5 of 75 (7%) patients (7 visits) in the intervention group and 6 of 75 (8%) patients (14 visits) in the control group (Table 5). Regarding acute outpatient visits for urological problems that resulted in unplanned admissions from outpatient to inpatient care, this occurred for 1 patient in the intervention group (1 admission) and 2 patients in the control group (3 admissions).

**Table 5:** Prostate cancer trial multivariate regression analysis of predictors for health care visits.

				Intervention group (n=75)	Control group (n=75)	B	SE	Standardized coefficient [Exp(B)]	95% CI	*P* value	*χ*^2^/*df*^a^
**Variables**											
	**Outpatient care visits**										
		Dependent variable: total visits, n		739	851	N/A^b^	N/A	N/A	N/A	.008^c^	.848
		Independent variables:									
			Group^d^	N/A	N/A	0.190	0.172	1.210	0.864-1.694	.27^e^	N/A
			Age	N/A	N/A	0.019	0.016	1.019	0.989-1.051	.22^f^	N/A
			HRQOL	N/A	N/A	–3.439	1.135	0.032	0.003-0.297	.002^f^	N/A
			Comorbidity	N/A	N/A	0.007	0.072	1.007	0.875-1.159	.92^f^	N/A
		Dependent variable: patients with acute visit, n		25	25					.88^f^	1.033
		Independent variables:									
			Group^g^	N/A	N/A	–0.017	0.350	0.983	0.495-1.953	.96^h^	N/A
			Age	N/A	N/A	0.017	0.030	1.017	0.959-1.079	.56^h^	N/A
			HRQOL	N/A	N/A	0.078	2.370	1.081	0.010-112.574	.97^h^	N/A
			Comorbidity	N/A	N/A	0.078	0.150	1.081	0.807-1.450	.60^h^	N/A
		Dependent variable: patients with acute visit for urological problems, n		5	6	N/A	N/A	N/A	N/A	.23^f^	1.048
		Independent variables:									
			Group^g^	N/A	N/A	–0.127	0.620	0.881	0.261-2.968	.84^h^	N/A
			Age	N/A	N/A	–0.022	0.057	0.978	0.875-1.093	.69^h^	N/A
			HRQOL	N/A	N/A	8.368	3.532	4304.883	4.239-4,371,830	.02^h^	N/A
			Comorbidity	N/A	N/A	0.142	0.284	1.152	0.661-2.008	.62^h^	N/A
		Dependent variable: patients with an unplanned admission, n		7	6	N/A	N/A	N/A	N/A	.43^f^	1.038
		Independent variables:									
			Group^g^	N/A	N/A	0.080	0.593	1.083	0.339-3.465	.89^h^	N/A
			Age	N/A	N/A	-0.069	0.060	0.933	0.830-1.049	.25^h^	N/A
			HRQOL	N/A	N/A	5.134	3.481	169.738	0.185-155,848.181	.14^h^	N/A
			Comorbidity	N/A	N/A	0.030	0.252	1.03	0.628-1.69	.90^h^	N/A
	**Inpatient care visits**										
		Dependent variable: patients with a visit, n		11	10					.12^f^	1.068
		Independent variables:									
			Group^g^	N/A	N/A	0.027	0.487	1.027	0.396-2.665	.96^h^	N/A
			Age	N/A	N/A	–0.065	0.049	0.937	0.852-1.030	.18^h^	N/A
			HRQOL	N/A	N/A	6.017	2.918	410.398	1.349-124,898.645	.04^h^	N/A
			Comorbidity	N/A	N/A	–0.064	0.198	0.938	0.637-1.382	.75^h^	N/A

^a^Pearson χ2 value divided by degrees of freedom (goodness of fit of the model).

^b^N/A: not applicable.

^c^*P* value Omnibus test General Linear Model Multivariate Regression.

^d^Intervention/Control; Reference category=Intervention

^e^*P* value for the independent variable in the General Linear Model Multivariate Regression.

^f^*P* value Omnibus test Binary Logistic Regression Model

^g^Intervention/Control; reference category=Control

^h^*P* value for the independent variable in the Binary Logistic Regression Model.

The mean inpatient cost was €1321 (SD €5460) in the intervention group, compared with €1049 (SD €4240) in the control group. In the intervention group, €1054 out of €1321 (79.79%) inpatient care costs were attributed to acute care, while the corresponding figure in the control group was €684 out of €1049 (65.20%). The most common diagnoses during acute inpatient care episodes in the intervention group were R060 dyspnea (4 episodes) and I214 acute subendocardial infarction (3 episodes). In the control group, the most common diagnoses were N390 urinary tract infection (3 episodes) and anemia D630/D649 (2 episodes).

Negative binomial multivariate regression analysis revealed that higher HRQOL before treatment was associated with a decrease in the number of outpatient care visits (*P*=.002). Multivariate binary logistic regression indicated that higher HRQOL before treatment was negatively associated with both having an acute outpatient care visit for urological problems (*P*=.02) and having an inpatient care episode (*P*=.04). Therefore, patients with better HRQOL before treatment were less likely to have an acute outpatient visit for urological problems and less likely to experience an inpatient care episode (Table 5).

## Discussion

### Principal Findings

In both RCTs, patients in the intervention groups experienced fewer QALYs lost compared with those in the control groups. In the B-RCT, the quality of life of patients in the intervention group decreased significantly less during treatment compared with the control group; however, this was not observed in the P-RCT. The reduction in QALYs lost was achieved with a low intervention cost per patient. Unfortunately, while ICERs are particularly valuable for guiding decision makers, the ICERs from this study are somewhat difficult to interpret due to uncertainty, as illustrated in the cost-effectiveness planes. The variability in the ICERs arose from differences in patients’ health care costs. When health care costs were excluded from the analysis, the RCTs showed ICERs slightly above the NBHW threshold for a low cost per QALY but still well below what the NBHW considers a high cost per QALY. These findings are encouraging and support the conceptual foundation of ePRO [48].

### Comparison With Prior Work

Although the P-RCT showed a decrease in QALYs lost, the difference was relatively small. This finding is not uncommon. Snoswell et al [49] reviewed 25 cost-utility studies of telehealth interventions that reported costs from the health system perspective and changes in HRQOL. About one-third of these studies demonstrated cost savings and changes in effect, but most QALY improvements were marginal (range 0.0006-0.12). The authors concluded that this may be partly due to HRQOL instruments being neither sensitive nor appropriate for detecting the effects of changes in health service delivery. Demonstrating substantial cost savings from ePRO during curative cancer treatment may be challenging, given the relatively short time frame and the high variability in health care utilization, which necessitate large sample sizes. However, because productivity loss due to morbidity and mortality represents the most significant societal cost of cancer [50], further research should explore whether life quality improvements enable patients to continue working during treatment or return to work earlier and to a greater extent after treatment. Such a cost-effectiveness analysis could reveal societal cost savings.

There are a few studies available to compare with our findings, as CUAs of ePRO interventions remain limited [27]. We did not identify any studies evaluating the cost-effectiveness of ePRO during first-line curative cancer treatment. However, some studies have been conducted in the context of follow-up and advanced cancer care. For instance, Nixon et al [51] and Lizée et al [28] reported relatively low ICERs for ePRO in cancer survivors, whereas Van der Hout et al [52] found a small positive effect on HRQOL but no significant differences in direct or indirect medical costs among cancer survivors.

Evidence suggests that ePRO can reduce health care utilization [27]. For example, ePRO has been shown to positively impact outcomes such as emergency room visits, hospitalizations, and readmissions [27]. However, not all studies demonstrate these effects. Barbera et al [53], in a study conducted during adjuvant chemotherapy for breast cancer, did not observe a reduction in hospitalizations or readmissions. Similarly, Wheelock et al [54] investigated the impact of ePRO during follow-up care after breast cancer treatment and found no reduction in health care resource use, including oncology-related appointments, physician visits, or medical tests. Lizée et al [28] observed a higher number of follow-up clinic visits in the ePRO intervention group compared with the control group. However, the intervention group also experienced longer overall survival, allowing more time for follow-up visits.

The study highlights the heterogeneity of the cancer population and the variation in health care use not only between but also within patient populations. For instance, nearly 104 of 149 (69.8%) patients treated for breast cancer made acute outpatient visits, whereas only about one-third (50/150) of patients with prostate cancer required such visits. This may be linked to the continuous health care contact that patients undergoing external RT maintain. In the context of older patients receiving cancer treatment, Nipp et al [55] demonstrated that age moderated the positive effects of ePRO on both ER visits and survival in patients with advanced cancer.

By analyzing ICD codes documented during health care visits to regional health care organizations, this study revealed that 5 of 75 (7%) intervention group patients and 6 of 75 (8%) control group patients undergoing treatment for prostate cancer had an acute outpatient visit for urological problems. Similarly, 4 of 74 (5%) intervention group patients and 11 of 75 (15%) control group patients treated for breast cancer had an acute outpatient visit for gastrointestinal symptoms. However, health care visits to general practitioners’ clinics and health centers lacked ICD codes, meaning these figures may not fully capture the patients’ health care utilization for those symptoms.

In this study, intervention group patients treated for breast cancer had more acute outpatient visits for fever/neutropenia, although the difference was not statistically significant. A similar increase in neutropenic events was observed by Absolom et al [10], who evaluated an intervention for patients undergoing chemotherapy, which aligns with national recommendations for managing chemotherapy patients presenting with this symptom [56]. In this study, the number of acute inpatient care episodes for fever/neutropenia was similar between the intervention and control groups.

Previous research has shown that health care utilization and costs during cancer treatment are complex [45]. This study examined whether age, comorbidity, and health status significantly influenced patients’ health care utilization. As expected, the results suggest that health status has some impact on health care consumption. In the B-RCT, higher HRQOL before treatment was associated with reduced acute outpatient costs. In the P-RCT, higher HRQOL before treatment was associated with a lower likelihood of having an acute outpatient visit or inpatient care episode. Considering the results showing a smaller decline in quality of life among intervention group patients, these findings suggest that patients using the app receive timely and appropriate care, leading to more effective and prompt management of symptoms and adverse events associated with cancer treatment. This interpretation aligns with previously reported positive effects on health-related quality of life and symptom burden [11,19,20].

### Limitations

The study presents unique and highly relevant findings for modern outpatient-based, personalized cancer care. A key strength is its randomized design, although some limitations should be noted. First, the ICERs must be interpreted with caution due to the uncertainty illustrated in the cost-effectiveness planes. Regarding costs, additional expenses for nurses handling alerts were not included, as staff interviews indicated that no increase in working hours was necessary (unpublished data). A similar assumption was made in the study by Nixon et al [51]. A recent study of a similar intervention also concluded that the intervention did not increase hospital clinicians’ workload [10]. Finally, the Swedish valuation system assigns higher values to most conditions than the British system, presumably because it is based on patients’ valuations of their conditions rather than, as in the British case, the public’s valuations of hypothetical conditions [57]. Accordingly, the results regarding intervention effectiveness may have differed.

In the P-RCT, the dropout rate was notably high, potentially reducing the statistical power to detect significant differences. The reasons for nonresponse to outcome questionnaires remain unknown. Importantly, all patients used the app daily as instructed, with an adherence rate of 80% [6]. Although debated, imputation aims to accurately estimate the overall data distribution [58]. It is suggested that imputing missing values exceeding 10% increases the risk of bias [34,58]. By contrast, the use of ITT analysis is highly recommended in RCTs [34]. Although there is no specific threshold for missing values in health economic studies, it is emphasized that patterns of missing data should be reported [32]. The sample in our study is too small to analyze patterns, but the ITT principle presumably assumes missing data are random, though other mechanisms may also have contributed [59]. Based on the study design and sample size, a simple imputation method was applied in this study [57].

Health care costs were missing at random due to an administrative error, and values for no more than 2 patients per group were imputed. The risk of overestimating costs due to right-skewed data is therefore small. Given that the data on the EORTC dimension scores are approximately normally distributed, the imputation method appears to be accurate. Nevertheless, further studies are needed.

### Conclusions

At a low weekly cost, the intervention reduced QALYs lost. The cost-effectiveness of the intervention, as defined by the ICER in relation to the Swedish NBHW, varied depending on the costs considered. For patients with breast cancer, the intervention was cost-effective when nonacute health care costs were excluded, whereas for patients with prostate cancer, cost-effectiveness was achieved when all health care costs were included. This suggests that the intervention has the potential to achieve cost-effectiveness. However, larger studies are needed, as there was considerable uncertainty regarding the ICERs due to significant variations in patients’ health care costs.

Patients in the intervention group with breast cancer had more acute health care visits for neutropenia/fever, whereas more patients in the control group were hospitalized for gastrointestinal symptoms. Only a few patients with prostate cancer were hospitalized for urological problems. These findings highlight the previously demonstrated positive effects on patients’ symptom burden and suggest that the intervention may facilitate timelier and more effective symptom management. Future studies should assess cost-effectiveness from a societal perspective.

## Appendix

Multimedia Appendix 1 CONSORT (Consolidated Standards of Reporting Trials) flowchart for the B-RCT. B-RCT: neoadjuvant chemotherapy for breast cancer-randomized controlled trial.

Multimedia Appendix 2 Cancer treatment B-RCT and P-RCT. B-RCT: neoadjuvant chemotherapy for breast cancer-randomized controlled trial; P-RCT: radiotherapy for prostate cancer-randomized controlled trial.

Multimedia Appendix 3 CONSORT (Consolidated Standards of Reporting Trials) flowchart P-RCT. P-RCT: radiotherapy for prostate cancer-randomized controlled trial.

Multimedia Appendix 4 P-RCT complete cases analysis. P-RCT: radiotherapy for prostate cancer-randomized controlled trial.

Multimedia Appendix 5 Sociodemographic and clinical characteristics.

Multimedia Appendix 6 The SIMKOST (simulerad kostnad/simulated cost) variable.

Multimedia Appendix 7 ICD codes categorized. ICD: International Statistical Classification of Diseases and Related Health Problems.

Multimedia Appendix 8 EQ-5DP dimensions.

Multimedia Appendix 9 CONSORT eHEALTH checklist (V 1.6.1).

## Abbreviations

B-RCT: neoadjuvant chemotherapy for breast cancer
CONSORT: Consolidated Standards of Reporting Trials
CUA: cost-utility analysis
ePRO: electronic patient-reported outcome
HRQOL: health-related quality of life
ICD: International Statistical Classification of Diseases and Related Health Problems
ICER: incremental cost-effectiveness ratio
ITT: intention-to-treat
NACT: neoadjuvant chemotherapy
NBHW: National Board of Health and Welfare
P-RCT: radiotherapy for prostate cancer
QALY: quality-adjusted life year
RT: radiotherapy
SEK: Swedish kronor

## Data Availability

The data sets generated and analyzed during this study are not publicly available as they contain information that could compromise the privacy and consent of the research participants. However, the transformed data are available upon reasonable request from the authors.
