# Supplementary figures and images for: Mobile Electronic Patient-Reported Outcomes and Interactive Support During Breast and Prostate Cancer Treatment: Health Economic Evaluation From Two Randomized Controlled Trials
Source: JMIR Cancer. 2025 Mar 11;11:e53539. doi: 10.2196/53539 (PMC11937708; doi:10.2196/53539)

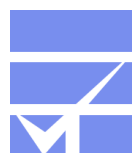

# CONSORT

TRANSPARENT REPORTING of TRIALS

## CONSORT B-RCT

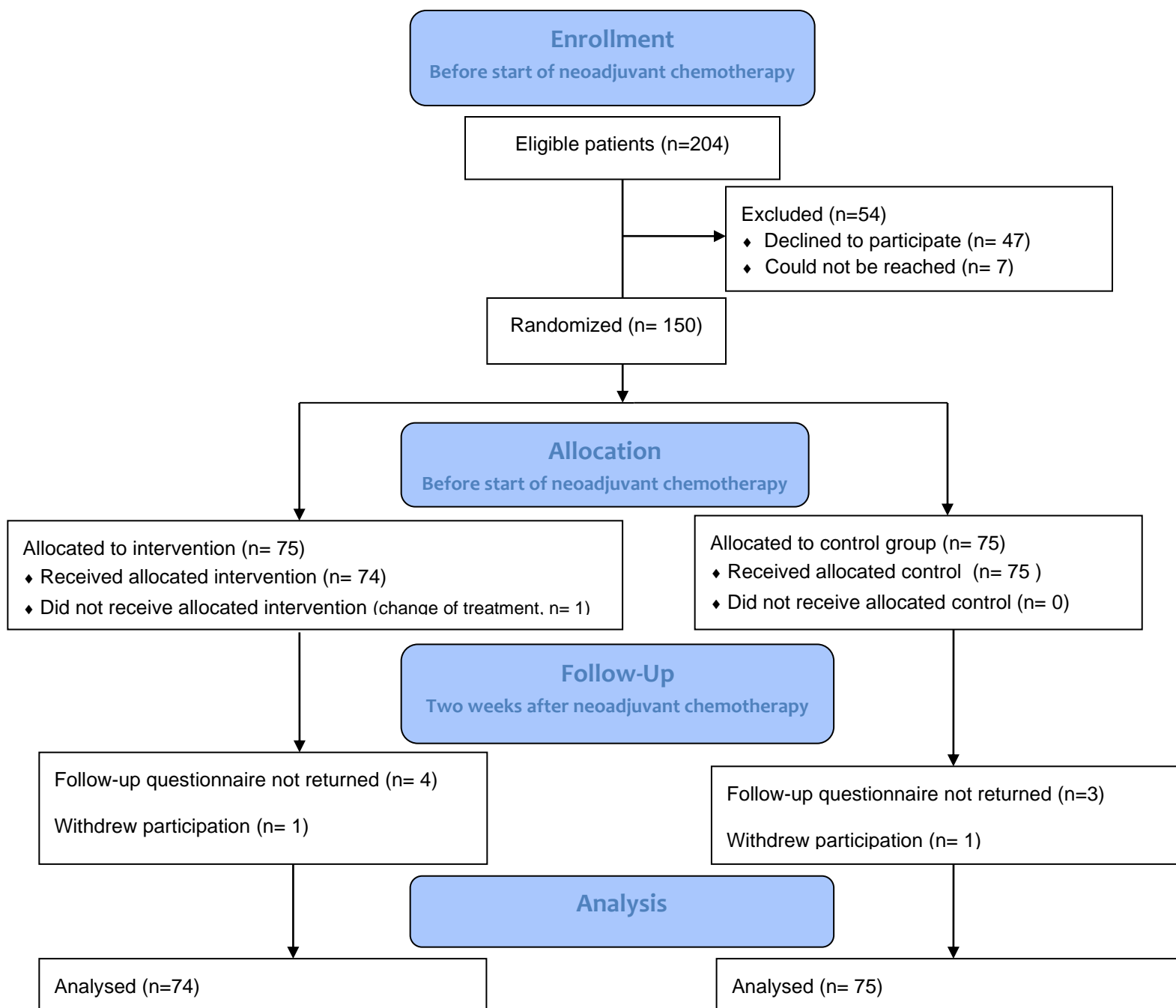

Supplement: Multimedia Appendix 1 [file cancer_v11i1e53539_app1.pdf]

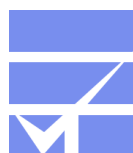

# CONSORT

TRANSPARENT REPORTING of TRIALS

## CONSORT P-RCT

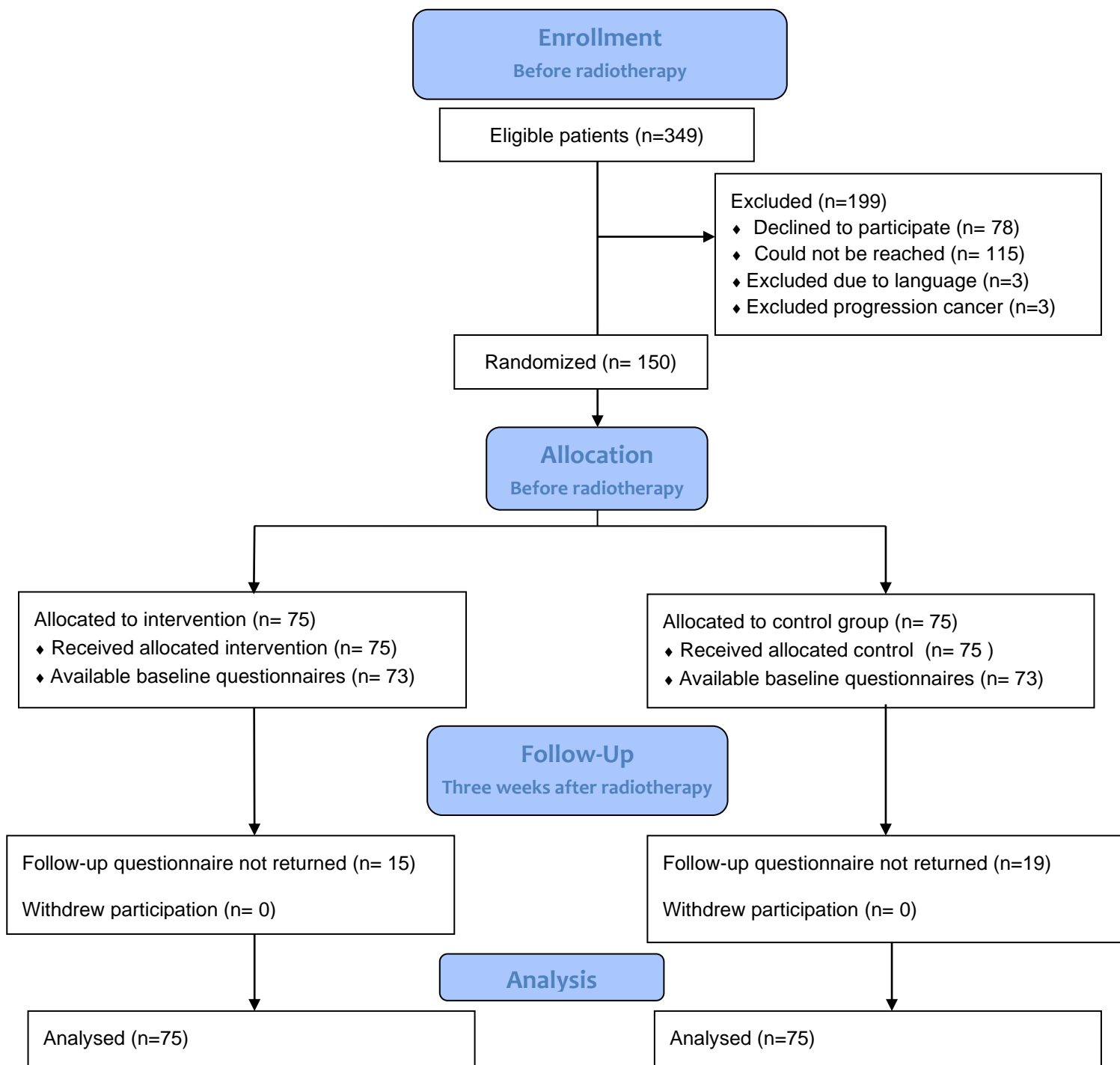

Supplement: Multimedia Appendix 3 [file cancer_v11i1e53539_app3.pdf]
