# Supplementary material for: Mobile Electronic Patient-Reported Outcomes and Interactive Support During Breast and Prostate Cancer Treatment: Health Economic Evaluation From Two Randomized Controlled Trials
Source: JMIR Cancer. 2025 Mar 11;11:e53539. doi: 10.2196/53539 (PMC11937708; doi:10.2196/53539)
Supplement: Multimedia Appendix 2 [file cancer_v11i1e53539_app2.docx]

| Breast cancer trial treatment | | |
| --- | --- | --- |
|  | Intervention group n=74 | Control group n=75 |
| **Number of NACT** |  |  |
| Mean (SD) | 6.9 (2.4) | 7.1 (2.8) |
| Median (Range) | 6.0 (2-15) | 6.0 (2-15) |
| **Treatment duration weeks** |  |  |
| Mean (SD) | 15 (2.6) | 15 (4.8) |
| **Completed NACT n (%)** |  |  |
| Yes | 65 (87.8) | 65 (86.7) |
| No | 9 (12.2) | 10 (13.3) |
| **Reasons not completed NACT** n (%) |  |  |
| Side-effects | 5 (55.6) | 3 (30.0) |
| Tumor progression | 2 (22.2) | 1 (10.0) |
| Generalized disease | 0 | 3 (30.0) |
| Side-effects and tumor progression | 0 | 3 (30.0) |
| Side-effects and generalized disease | 1, (11.1) | 0 |
| No tumor response and generalized disease | 1, (11.1) | 0 |
| **Type of chemotherapy** n (%) |  |  |
| Anthracyclines, Alkylators | 3 (4.1) | 1 (1.3) |
| Anthracyclines, Alkylators, Taxanes | 37 (50.0) | 38 (50.7) |
| Anthracyclines, Alkylators, Antimetabolites, Taxanes | 9 (12.2) | 13 (17.3) |
| Antimetabolites, Alkylators | 2 (2.7) | 1 (1.3) |
| Taxanes | 11 (14.9) | 14 (18.7) |
| Taxanes, Alkylators | 1 (1.4) | 0 |
| Taxanes, T-DM1 | 2 (2.7) | 2 (2.7) |
| T-DM1 | 9 (12.2) | 6 (8.0) |
| **Antibody treatment combined with NACT** n (%) |  |  |
| No | 45 (60.8) | 4 (58.7) |
| Yes | 29 (39.2) | 31 (41.3) |
| **Type of antibody treatment** n (%) |  |  |
| Trastuzumab and Pertuzumab | 20 (69.0) | 25 (80.6) |
| Trastuzumab | (31.0) | 6 (19.4) |
| **Died from breast cancer 43 months after randomization** n (%) |  |  |
| No | 68 (92) | 69 (92) |
| Yes | 6 (8) | 6 (8) |
| **Died from other disease 43 months after randomization** n (%) |  |  |
| No | 74 (100) | 75 (100) |
| Yes | 0 | 0 |

| Prostate cancer trial treatment | | |
| --- | --- | --- |
|  | Intervention group n=75 | Control group n=75 |
| **Radiotherapy treatment regimen** |  |  |
| 25 external + 2 internal (brachytherapy) n, (%) | 37, (49) | 37, (49) |
| 29 external | 38, (51) | 36 |
| Other |  | 2* |
| **External treatment duration, weeks** Mean, (SD) | 5.39, (.40) | 5.37, (.42) |
| **Androgen deprivation therapy during treatment n, (%)** |  |  |
| No | 24, (32) | 21, (28) |
| Yes | 51, (68) | 54, (72) |
| **Died from prostate cancer 43 months after randomization** n, (%) |  |  |
| No | 75, (100) | 75, (100) |
| Yes | 0, (0) | 0, (0) |
| **Died from other disease 43 months after randomization** n, (%) |  |  |
| No | 72, (96) | 69, (92) |
| Yes | 3, (4) | 6, (8) |
| * One patient received 25 ext. RT and one patient 21 ext. RT | | |
