# Supplementary material for: Mobile Electronic Patient-Reported Outcomes and Interactive Support During Breast and Prostate Cancer Treatment: Health Economic Evaluation From Two Randomized Controlled Trials
Source: JMIR Cancer. 2025 Mar 11;11:e53539. doi: 10.2196/53539 (PMC11937708; doi:10.2196/53539)
Supplement: Multimedia Appendix 4 [file cancer_v11i1e53539_app4.docx]

**Supplementary file 3.**

| **P-RCT Complete cases analysis mean health-related quality-of-life changes in EORTC-QLQ-C30 from before radiotherapy treatment (baseline) until three weeks after (follow-up)** | | | | | | | |
| --- | --- | --- | --- | --- | --- | --- | --- |
|  | Intervention (n = 58) | | Control (n = 55) | |  | | |
|  | Mean | *(SD)* | Mean | *(SD)* | *t* | df | *p* |
| *Functioning scales change^1^* |  |  |  |  |  |  |  |
| Physical functioning | 5.17 | *(11.91)* | 3.50 | *(11.93)* | *.75* | 111 | .454 |
| Role functioning | -.57 | *(28.95)* | 3.09 | *(25.30)* | *-.71* | 110 | .479 |
| Emotional functioning | -3.30 | *(18.13)* | -1.16 | *(14.89)* | *-.69* | 111 | .495 |
| Cognitive functioning | -1.44 | *(14.74)* | -0.61 | *(13.59)* | *-.31* | 111 | .756 |
| Social functioning | 10.63 | *(26.25)* | 4.55 | *(25.35)* | *1.25* | 111 | .213 |
| *Symptom scales change^2^* |  |  |  |  |  |  |  |
| Fatigue | -6.51 | *(20.61)* | -7.47 | *(16.29)* | *.27* | 111 | .785 |
| Nausea and vomiting | -3.16 | *(10.10)* | -3.64 | *(9.46)* | *.26* | 111 | .797 |
| Pain change | .86 | *(21.04)* | -1.54 | *(21.54)* | *.60* | 110 | .551 |
| Dyspnoea | .57 | *(22.93)* | -1.82 | *(24.36)* | *.54* | 111 | .592 |
| Insomnia | 1.17 | *(29.52)* | .62 | *(30.71)* | *.10* | 109 | .923 |
| Appetite loss | -.57 | *(24.58)* | -3.64 | *(17.77)* | *.76* | 111 | .452 |
| Constipation | .57 | *(23.77)* | 1.82 | *(17.47)* | *-.32* | 111 | .753 |
| Diarrhoea | -2.87 | *(23.60)* | -12.12 | *(25.14)* | *2.01* | 110 | .047* |
| Financial difficulties | -2.87 | *(12.92)* | -3.70 | *(10,57)* | *.37* | 108 | .71* |
| Global health status /QoL | 5.46 | *(18.11)* | 7.58 | *(12,66)* | *-.72* | 102 | .471* |
| ^1^ A higher value indicates a decrease in score and a decreased function.  ^2^ A higher value indicates a decrease in scores and a decreased symptom burden.  * Equal variances not assumed | | | | | | | |
