# Supplementary material for: Mobile Electronic Patient-Reported Outcomes and Interactive Support During Breast and Prostate Cancer Treatment: Health Economic Evaluation From Two Randomized Controlled Trials
Source: JMIR Cancer. 2025 Mar 11;11:e53539. doi: 10.2196/53539 (PMC11937708; doi:10.2196/53539)
Supplement: Multimedia Appendix 5 [file cancer_v11i1e53539_app5.docx]

| **Patient self-reported sociodemographic data and pre-treatment clinical characteristics from medical journal** | | | | | | | | |
| --- | --- | --- | --- | --- | --- | --- | --- | --- |
|  | **Breast cancer trial (B-RCT)** | | | | **Prostate cancer trial (P-RCT)** | | | |
|  | Intervention n=74 | | Control n=75 | | Intervention n=75 | | Control n=75 | |
| **Age at inclusion, years** Mean (SD) | 48 | (10.6) | 50 | (11.6) | 70 | (6.7) | 70 | (5.6) |
| **Marital status** n (%) |  |  |  |  |  |  |  |  |
| Married / Cohabitating | 61 | (82.4) | 57 | (76.0) | 54 | (73.9) | 55 | (75.3) |
| Living alone | 13 | (17.6) | 18 | (24.0) | 17 | (23.2) | 16 | (21.9) |
| Missing data |  |  |  |  | 2 | (2.7) | 2 | (2.7) |
| **Education level** n (%) |  |  |  |  |  |  |  |  |
| University | 50 | (67.6) | 44 | (58.7) | 30 | (40) | 24 | (32.0) |
| Not university | 24 | (32.4) | 31 | (41.3) | 41 | (54.6) | 47 | (71.7) |
| Missing data |  |  |  |  | 2 | (2.7) | 2 | (2.7) |
| **Occupation** n (%) |  |  |  |  |  |  |  |  |
| Working | 57 | (77.0) | 48 | (64.0) | 22 | (30.1) | 20 | (27.3) |
| Not working* | 17 | (23.0) | 27 | (36.0) | 49 | (67.0) | 51 | (69.8) |
| Missing data |  |  |  |  | 2 | (2.7) | 2 | (2.7) |
| **Medical history of conditions** n (%) |  |  |  |  |  |  |  |  |
| Cardiovascular | 2 | (3) | 0 | (0) | 9 | (12) | 18 | (24) |
| Respiratory | 2 | (3) | 3 | (4) | 16 | (21) | 9 | (12) |
| Joint problems | 9 | (12) | 5 | (7) | 19 | (25) | 9 | (12) |
| Diabetes | 1 | (1) | 0 | (0) | 10 | (13) | 10 | (13) |
| Gastrointestinal | 12 | (16) | 7 | (9) | 10 | (13) | 6 | (8) |
| Cancer | 6 | (8) | 5 | (7) | 13 | (17) | 8 | (11) |
| Renal or Urological | 0 | (0) | 1 | (1) | 6 | (8) | 7 | (9) |
| Neurological | 4 | (5) | 8 | (11) | 3 | (4) | 3 | (4) |
| Thyroid problems | 6 | (8) | 7 | (9) | 3 | (4) | 1 | (1) |
| Mental health | 2 | (3) | 3 | (4) | 2 | (3) | 1 | (1) |
| **Menstruation status** n (%) |  |  |  |  |  |  |  |  |
| Premenopausal | 45 | (60.8) | 41 | (54.7) |  |  |  |  |
| Postmenopausal | 28 | (37.8) | 31 | (41.3) |  |  |  |  |
| Missing data | 1 | (1.4) | 3 | (4.0) |  |  |  |  |
| **Side of breast cancer** n (%) |  |  |  |  |  |  |  |  |
| Left | 39 | (52.7) | 43 | (57.3) |  |  |  |  |
| Right | 35 | (47.3) | 30 | (40) |  |  |  |  |
| Bilateral | 0 | (0) | 2 | (2.7) |  |  |  |  |
| **Her2 amplified** n (%) |  |  |  |  |  |  |  |  |
| Negative | 45 | (60.8) | 44 | (58.7) |  |  |  |  |
| Positive | 29 | (39.2) | 31 | (41.3) |  |  |  |  |
| Estrogen receptor (ER), Mean (SD) | 44.4 | (45.8) | 54.6 | (44.8) |  |  |  |  |
| Progesterone receptor (PR), Mean (SD) | 24.6 | (35.9) | 30.0 | (38.1) |  |  |  |  |
| Proliferation rate (Ki-67), Mean (SD) | 57.3 | (22.5) | 46.0 | (21.1) |  |  |  |  |
| **Tumour characteristics** n (%) |  |  |  |  |  |  |  |  |
| Her2+ ER+ PR+ | 9 | (12.2) | 13 | (17.3) |  |  |  |  |
| Her2+ ER+ PR- | 7 | (9.5) | 8 | (10.7) |  |  |  |  |
| Her2+ ER- PR- | 13 | (17.6) | 10 | (13.3) |  |  |  |  |
| Her2- ER+ PR+ | 16 | (21.6) | 20 | (26.7) |  |  |  |  |
| Her2- ER+ PR- | 7 | (9.5) | 8 | (10.7) |  |  |  |  |
| Her2- ER- PR+ | 1 | (1.4) | 0 |  |  |  |  |  |
| Triple negative | 21 | (28.4) | 16 | (21.3) |  |  |  |  |
| **Histologic grade (Elston-Ellis)** n (%) |  |  |  |  |  |  |  |  |
| Intermediate grade 2 | 23 | (31.1) | 29 | (38.7) |  |  |  |  |
| High grade 3 | 41 | (55.4) | 31 | (41.3) |  |  |  |  |
| Unknown | 10 | (13.5) | 15 | (20.0) |  |  |  |  |
| **Axillary lymph nodes** n (%) |  |  |  |  |  |  |  |  |
| Yes | 45 | (60.8) | 54 | (72.0) |  |  |  |  |
| No | 29 | (39.2) | 21 | (28.0) |  |  |  |  |
| **Type of tumour** n (%) |  |  |  |  |  |  |  |  |
| Ductal | 68 | (91.9) | 65 | (86.7) |  |  |  |  |
| Lobular | 2 | (2.7) | 3 | (4.0) |  |  |  |  |
| Mucinous | 1 | (1.4) | 2 | (2.7) |  |  |  |  |
| Medullary | 2 | (2.7) | 0 |  |  |  |  |  |
| Papillary | 0 |  | 1 | (1.3) |  |  |  |  |
| Metaplastic | 0 |  | 1 | (1.3) |  |  |  |  |
| Unknown | 1 | (1.4) | 3 | (4.0) |  |  |  |  |
| **PSA** Mean (SD) |  |  |  |  | 8.3 | (10.4) | 7.8 | (11.4) |
| **Gleason** Mean (SD) |  |  |  |  | 7.4 | (.79) | 7.3 | (.83) |
| **Cancer stage** n (%) |  |  |  |  |  |  |  |  |
| T1 |  |  |  |  | 1 | (1) | 0 | (0) |
| T1C |  |  |  |  | 17 | (23) | 17 | (23) |
| T2 |  |  |  |  | 11 | (15) | 15 | (20) |
| T2/T3 |  |  |  |  | 0 | (0) | 2 | (3) |
| T2A |  |  |  |  | 1 | (1) | 1 | (1) |
| T2B |  |  |  |  | 7 | (9) | 1 | (1) |
| T2C |  |  |  |  | 7 | (9) | 8 | (11) |
| T3 |  |  |  |  | 20 | (27) | 23 | (31) |
| T3A |  |  |  |  | 1 | (1) | 1 | (1) |
| T3B |  |  |  |  | 8 | (11) | 6 | (8) |
| Missing data |  |  |  |  | 2 | (3) | 1 | (1) |
| *(sick leave / retired / unemployed) | | | | | | | | |
