# Supplementary material for: Mobile Electronic Patient-Reported Outcomes and Interactive Support During Breast and Prostate Cancer Treatment: Health Economic Evaluation From Two Randomized Controlled Trials
Source: JMIR Cancer. 2025 Mar 11;11:e53539. doi: 10.2196/53539 (PMC11937708; doi:10.2196/53539)
Supplement: Multimedia Appendix 6 [file cancer_v11i1e53539_app6.docx]

**Calculation methods for the variable SIMKOST**

**Background**

The Stockholm County Council VAL Databases for Outpatient Care (OVR) and Inpatient Care (SLV) hold the Stockholm County council residents’ visits and costs for healthcare. The costs are described in the variable Totkost (Total cost). The degree to which Totkost captures the county council's real costs depends, among other things, on which reimbursement models are applied for the different types of assignments included in each branch of care. The best agreement is found when it comes to acute somatic care, where Totkost is based on diagnosis-related groups (Nord DRG) whose weights are in turn based on cost per patient (KPP).

To calculate SIMKOST we

1. Deducted the county council's costs for private specialists on national rates or care agreements according to the ARV database from the profit and loss account and per economic care branch.  *"income statement" hereafter means the income statement adjusted in the aforementioned manner.*
2. Compare the summed Totkost in OVR and SLV per economic branch of care against HSF's income statement per branch of care.
3. Compare the summed Totkost in OVR and SLV per economic branch of care against HSF's income statement per branch of care.
4. Recalculated and added to the existing Totkost to reach full agreement with the amounts in the profit and loss accounts.
5. In the absence of appropriate distribution keys, we have not included the costs reported in the income statement for "Customers' own operations" (currently around 0.7 billion per year) and for "Medicines in open care" (currently around 5 billion per year).

**Acute somatic care**

Summed Totkost for acute somatic care is close to the income statement amount. In order to obtain Simkost, we therefore only add up Totkost to full agreement with the amount in the profit and loss account.

**Geriatrics**

The inconsistency between summed Totkost and the income statement for this branch of care is primarily due to the absence of Totkost for ASIH (community nursing) in OVR. We supplement Totkost with the county council's average visit cost for ASIH, which we obtain by dividing the cost of ASIH according to DEBval (the invoicing system HEJ) by the total number of ASIH visits according to OVR. To keep the calculation simple and repeatable, we do not differentiate between different visit and assignment types. We enter the amounts and make a new summation of Totkost. In order to arrive at Simkost, we finally add up Totkost to fully agree with the income statement amount.

**Primary care**

Totkost is highly dependent on the distribution between visit reimbursement and capitation applicable for each year and, in addition, is too incomplete to, unlike acute somatic care, hold for enumeration. To calculate Simkost, we instead use the relative weight between a doctor's visit and another visit according to the National Board of Health and Welfare's "Care in numbers, cost per care contact in primary care", where the cost of a visit to a staff category other than a doctor is calculated to correspond to 40% of a doctor's visit. We divide the income statement amount by the volumes weighted in this way in OVR for doctor's visits and other visits. To keep the calculation simple and repeatable, we do not differentiate between different visit and assignment types. The approach provides, for mathematical reasons, full agreement between summed Simkost and the amount in the profit and loss account.

**Psychiatry**

Totkost, like primary care, is strongly dependent on current forms of compensation and is too uneven and fragmentary to count. Instead, we use the average cost for a visit and a day of care according to SKL's KPP to calculate the relative weight between a visit and a day of care. We divide the amount of the income statement by the visitor and spring day volumes weighted in this way in OVR and SLV respectively. To keep the calculation simple and repeatable, we do not differentiate between different visit and assignment types. The approach provides, for mathematical reasons, full agreement between summed Simkost and the amount in the profit and loss account.

**Other healthcare**

With regard to other healthcare, the difference between the totaled Totkost and the amount in the profit and loss account is mainly due to the fact that Totkost is missing for certain visit types. We calculate Simkost by dividing the number of visits that lack Totkost by the difference. For the sake of simplicity, we do not differentiate between different visit or mission types. The approach provides, for mathematical reasons, full agreement between summed Simkost and the amount in the profit and loss account.

Contact person: Göran Lord, Hsf (Region Stockholm)
