# Supplementary material for: Mobile Electronic Patient-Reported Outcomes and Interactive Support During Breast and Prostate Cancer Treatment: Health Economic Evaluation From Two Randomized Controlled Trials
Source: JMIR Cancer. 2025 Mar 11;11:e53539. doi: 10.2196/53539 (PMC11937708; doi:10.2196/53539)
Supplement: Multimedia Appendix 7 [file cancer_v11i1e53539_app7.pdf]

Categorized International Classification of Diseases (ICD) codes

**B-RCT**	**Diagnose text**	
D709C	Neutropenia UNS	**Fever/Neutropenia**
R502	Drug-induced fever	
R508	Other specified fever	
R509	Fever, unspecified	
K521	Toxic gastroenteritis and colitis	**Gastroenteritis, colitis**
A047	Enterocolitis caused by Clostridium difficile	
D649	Anaemia, unspecified	**Anaemia**
N390	Urinary tract infection, unspecified location	**Urinary tract infection**
**P-RCT**		
R339	Urinary retention (urinary incontinence)	**Urinary problems**
N390	Urinary tract infection, unspecified location	
R301	Tenesmus in the bladder	
N390X	Urinary tract infection, unspecified location	
N304	Radiation cystitis	
N300	Acute cystitis	
R391	Other micturition difficulties	
N109	Acute tubulo-interstitial nephritis	
T830	Mechanical complication of quaternary catheter	
R319	Unspecified hematuria	
