# Supplementary material for: Mobile Electronic Patient-Reported Outcomes and Interactive Support During Breast and Prostate Cancer Treatment: Health Economic Evaluation From Two Randomized Controlled Trials
Source: JMIR Cancer. 2025 Mar 11;11:e53539. doi: 10.2196/53539 (PMC11937708; doi:10.2196/53539)
Supplement: Multimedia Appendix 8 [file cancer_v11i1e53539_app8.docx]

**Supplementary file EQ-5DP Dimensions**

| **B-RCT Patients EQ-5DP dimension levels before and after treatment** | | | | | | | | | | | | | | | | | | | | |
| --- | --- | --- | --- | --- | --- | --- | --- | --- | --- | --- | --- | --- | --- | --- | --- | --- | --- | --- | --- | --- |
|  | **Mobility** | | | | **Self-care** | | | | **Usual Activities** | | | | **Pain/Discomfort** | | | | **Anxiety/Depression** | | | |
|  | IG | | CG | | IG | | CG | | IG | | CG | | IG | | CG | | IG | | CG | |
|  | BL | FU | BL | FU | BL | FU | BL | FU | BL | FU | BL | FU | BL | FU | BL | FU | BL | FU | BL | FU |
| **Level 1** n | 66 | 55 | 67 | 52 | 72 | 69 | 73 | 66 | 46 | 30 | 51 | 27 | 29 | 27 | 31 | 23 | 39 | 47 | 38 | 38 |
| **Level 2** n | 8 | 19 | 8 | 22 | 2 | 5 | 2 | 9 | 27 | 40 | 23 | 41 | 43 | 45 | 43 | 47 | 30 | 24 | 30 | 31 |
| **Level 3** n | 0 | 0 | 0 | 1 | 0 | 0 | 0 | 0 | 1 | 4 | 1 | 7 | 2 | 2 | 1 | 5 | 5 | 3 | 7 | 6 |
| **Some Problems*** n | 8 | 19 | 8 | 23 | 2 | 5 | 2 | 9 | 28 | 44 | 24 | 48 | 45 | 47 | 44 | 52 | 35 | 27 | 37 | 37 |
| Change reporting some problems** n (%) | 11 (15) | | 15 (20) | | 3 (4) | | 7 (9) | | 16 (22) | | 24 (32) | | 2 (3) | | 8 (11) | | -8 (-11) | | 0 (0) | |
| Difference in change*** (%) | (-5) | |  | | (-5) | |  | | (-10) | |  | | (-8) | |  | | (-11) | |  | |
| Total difference in change (%) | (-39) | |  | |  | |  | |  | |  | |  | |  | |  | |  | |
| BL=Baseline/Before treatment FU=Follow-up/After treatment IG=Intervention group CG=Control group  * Level 2+3  ** Follow-up - Baseline  *** Intervention group – Control group | | | | | | | | | | | | | | | | | | | | |

| **P-RCT Patients EQ-5DP dimension levels before and after treatment** | | | | | | | | | | | | | | | | | | | | | |
| --- | --- | --- | --- | --- | --- | --- | --- | --- | --- | --- | --- | --- | --- | --- | --- | --- | --- | --- | --- | --- | --- |
|  | **Mobility** | | | | **Self-care** | | | | **Usual activities** | | | | **Pain/Discomfort** | | | | **Anxiety/Depression** | | | | |
|  | IG | | CG | | IG | | CG | | IG | | CG | | IG | | CG | | IG | | | CG | |
|  | BL | FU | BL | FU | BL | FU | BL | FU | BL | FU | BL | FU | BL | FU | BL | FU | BL | FU | BL | | FU |
| **Level 1** n | 55 | 52 | 57 | 55 | 69 | 69 | 71 | 70 | 51 | 44 | 55 | 49 | 32 | 31 | 37 | 33 | 50 | 55 | 49 | | 52 |
| **Level 2** n | 19 | 23 | 18 | 20 | 6 | 6 | 4 | 5 | 22 | 29 | 19 | 25 | 41 | 42 | 37 | 41 | 24 | 19 | 24 | | 22 |
| **Level 3** n | 1 | 0 | 0 | 0 | 0 | 0 | 0 | 0 | 2 | 2 | 1 | 1 | 2 | 2 | 1 | 1 | 1 | 1 | 2 | | 1 |
| Some problems* n | 20 | 23 | 18 | 20 | 6 | 6 | 4 | 5 | 24 | 31 | 20 | 26 | 43 | 44 | 38 | 42 | 25 | 20 | 26 | | 23 |
| Change reporting some problems** n (%) | 3 (4) | | 2 (3) | | 0 (0) | | 1 (1) | | 7 (9) | | 6 (8) | | 1 (1) | | 4 (5) | | -5 (-7) | | | -3 (-4) | |
| Difference in change*** (%) | (1) | |  | | (-1) | |  | | (1) | |  | | (-4) | |  | | (-3) | | |  | |
| Total difference in change (%) | -6 |  |  |  |  |  |  |  |  |  |  |  |  |  |  |  |  |  |  | |  |
| BL=Baseline/Before treatment FU=Follow-up/After treatment IG=Intervention group CG=Control group  * Level 2+3  ** Follow-up - Baseline  *** Intervention group – Control group | | | | | | | | | | | | | | | | | | | | | |
